# Design and Development of Teixobactin Analog-Loaded Magnetic Nanocomposites for Biofilm Destruction and Pathogen Elimination

**DOI:** 10.3390/jfb17040189

**Published:** 2026-04-13

**Authors:** Huaxiang Lei, Ye Liang, Xuechen Li, Xiaojing Huang, Chengfei Zhang, Ting Zou

**Affiliations:** 1 Restorative Dental Sciences, Faculty of Dentistry, The University of Hong Kong, Hong Kong SAR, China; 2 Fujian Key Laboratory of Oral Diseases & Fujian Provincial Engineering Research Center of Oral Biomaterial & Clinical Research Center for Oral Tissue Deficiency Diseases of Fujian Province, School and Hospital of Stomatology, Fujian Medical University, Fuzhou 350001, China; 3 Department of Chemistry, State Key Laboratory of Synthetic Chemistry, The University of Hong Kong, Hong Kong SAR, China; 4 Shenzhen Clinical College of Stomatology, School of Stomatology & Shenzhen Stomatology Hospital (Pingshan), Southern Medical University, Shenzhen 518118, China

**Keywords:** biofilm eradication, EPS, _L_-Chg_10_-teixobactin, magnetic nanoparticles, rhamnolipid

## Abstract

Although teixobactin, a promising cyclic undecadepsipeptide, exhibits efficacy against Gram-positive bacteria due to its novel mode of action and low potential for resistance, its clinical application is limited by two key shortcomings: ineffectiveness against Gram-negative bacteria and poor penetration of the protective extracellular polymeric substance (EPS) in biofilms. This renders it unsuitable for targeting the polymicrobial biofilms, which are the cause of periodontitis and peri-implantitis. We designed a modified teixobactin analog by integrating rhamnolipid, Ag@Fe_3_O_4_ nanoparticles, and _L_-Chg_10_-teixobactin to obtain a novel magnetic nanoparticle (MNP). The MNP demonstrates the ability to simultaneously degrade EPS, penetrate biofilm structures, and eliminate both G^+^ and G^−^ pathogens under a rotating magnetic field (RMF). Rhamnolipid grafting degraded 52.5% of biofilm EPS. MNPs showed broad-spectrum antimicrobial activity, with minimal inhibitory concentrations from 100 to 200 µg/mL. Combined with RMF, biofilm eradication rates reached 97.0% (*E. faecalis*), 97.7% (*S. gordonii*), 88.4% (*P. gingivalis*), and 74.2% (*F. nucleatum*). The biofilm thickness was reduced from 19.4 ± 2.9 µm to 7.4 ± 1.0 µm, and the biofilm biomass was reduced by 68.5%. This combined strategy integrates enzymatic EPS degradation, magneto-mechanical disruption, and dual antimicrobial action, offering a promising topical therapy for periodontitis and peri-implantitis.

## 1. Introduction

Antimicrobial resistance commonly emerges within the intricate extracellular polymeric substance (EPS) of biofilms, which form on diverse surfaces including teeth, mucosal tissues, and medical devices, entrapping both Gram-positive (G^+^) pathogens and Gram-negative (G^−^) pathogens within their 3D framework [1,2]. Such unique physical and metabolic barriers not only hinder the actions of the host immune system but also impede the penetration and efficacy of antimicrobial agents [3,4]. Therefore, the development of highly efficient novel antimicrobial agents that are less likely to induce resistance, along with the construction of delivery systems capable of effectively penetrating the bacterial biofilm barrier, is of great importance for improving the treatment outcomes of infectious diseases.

Researchers have explored various alternative therapeutic strategies to combat bio-film-associated infections. Ionic liquids (ILs), classified as a type of tunable “designer solvents,” have gained considerable attention in the antimicrobial field due to their structural similarity to cationic biocides and their membrane-disrupting capabilities [5,6,7,8]. Another innovative strategy involves the development of silver-phage conjugates, which are nanohybrids that integrate the targeted penetration capability of bacteriophages with the broad-spectrum antibacterial effects of silver nanoparticles to create a highly efficient synergistic platform [9]. Meanwhile, antimicrobial peptides are considered potential alternatives to traditional antibiotics due to their unique mechanisms of action and low propensity to induce resistance. Among them, demonstrating excellent activity against G^+^ bacteria by specifically binding to the cell wall precursors lipid II and lipid III to achieve efficient bactericidal effects [10]. Particularly important is that teixobactin targets highly conserved regions of bacterial cell wall precursor molecules that are essential for bacterial survival, making it difficult for bacteria to develop resistance by altering these targets, thereby exhibiting an extremely low potential for resistance induction [11]. However, due to the obstruction posed by the outer membrane structure of G^−^ bacteria, teixobactin’s inhibitory effect against G^−^ pathogens is limited, which severely restricts its clinical application and prompts researchers to explore combination therapy strategies [12].

Although silver nanoparticles exhibit antimicrobial activity against both G^+^ and G^−^ bacteria, their efficacy and mechanisms of action differ significantly between these two classes. Ag^+^ demonstrates enhanced potency against G^−^ bacteria because of its preferential interaction with phospholipids and lipopolysaccharides in the outer membrane. Conversely, its activity against G^+^ bacteria is relatively limited since the thick peptidoglycan layer restricts Ag^+^ penetration [13]. In contrast, teixobactin and its analogs show remarkable potency against G^+^ bacteria [12]. Given this limitation, silver ions (Ag^+^) and their nanoparticles (AgNPs)—as classic antimicrobial agents—hold considerable value as a complementary therapeutic strategy. They exhibit significantly enhanced antimicrobial activity against G^−^ bacteria [13,14], effectively compensating for teixobactin’s limited antimicrobial spectrum. The antimicrobial mechanisms of silver ions are diverse: besides disrupting cell membrane integrity, Ag^+^ can also electrostatically interact with DNA bases in cationic form, destroying the DNA double helix structure, thereby achieving efficient bactericidal effects [14].

Although both teixobactin analogs and silver nanomaterials possess significant bactericidal potential, their efficacy is severely limited by poor penetrability through the EPS-based biofilm architecture. Within these intricate EPS-entrapped networks—where both G^+^ and G^−^ pathogens coexist—multidrug resistance readily emerges. This is particularly evident in oral biofilms, which typically exist as polymicrobial communities composed of both G^+^ and G^−^ bacteria, such as the transitional mediator G^−^ *Fusobacterium nucleatum,* early colonizers like G^+^ *Streptococcus gordonii,* and late colonizers such as G^−^ *Porphyromonas gingivalis* [15]. These polymicrobial biofilms are major contributors to oral infectious diseases like periodontitis and peri-implantitis. To enhance biofilm eradication, concerted research efforts have explored various strategies to disrupt biofilm integrity. Chemical methods primarily achieve degradation by targeting fundamental EPS components such as lipids, DNA, polysaccharides, and proteins [16]; physical methods include constructing biohybrid systems integrating magnetotactic bacteria with silica microtubules [17], or using antimicrobial magnetic nanoparticles (MNPs) to form penetration channels within biofilms [18]. However, chemical strategies face challenges, including the ineffectiveness of single components against the diverse composition of EPS, the time-consuming nature of enzymatic hydrolysis, and limited diffusion distances [19]; physical methods are constrained by insufficient disruption of biofilm structure and limited penetration capability within the biofilm matrix [20]. Consequently, there is an urgent need to develop novel integrated strategies that can synergistically overcome these limitations.

Based on this rationale, this study designed rhamnolipid (RL)-functionalized Ag@Fe_3_O_4_ MNPs that combine chemical modification and physical actuation to enhance anti-biofilm efficacy. RL, a naturally occurring glycolipid surfactant, degrades proteins and DNA within the biofilm matrix and interacts with extracellular polysaccharides, thereby softening and dispersing the biofilm structure to promote the penetration of antimicrobial components [21]. The Ag@Fe_3_O_4_ core–shell structure not only provides magnetic responsiveness, enabling directional movement and enhanced biofilm penetration under the guidance of a rotating magnetic field (RMF), but also incorporates silver ions (Ag^+^) antimicrobial activity, compensating for teixobactin analogs’ limited efficacy against G^−^ bacteria.

**Scheme 1:**
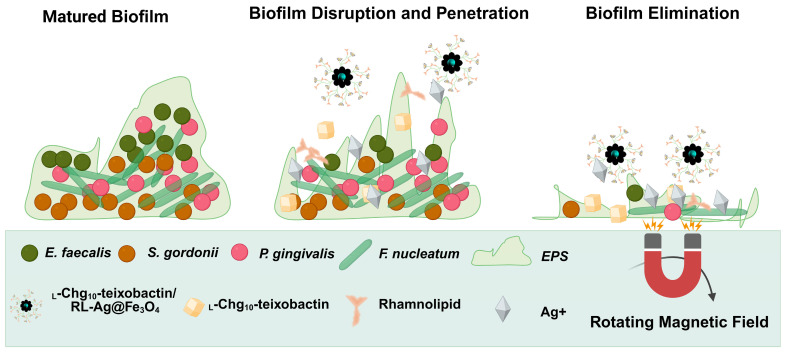
The _L_-Chg_10_-teixobactin/RL-Ag@Fe_3_O_4_ MNPs combine the EPS-degrading RL with the mechanical forces applied by an external magnetic field to disassemble the matrix scaffold, exposing the bacterial cells. When incorporated into one MNP, two antimicrobial composites, Ag^+^ and _L_-Chg_10_-teixobactin, which target G^+^ and G^−^ bacteria, respectively, can effectively enhance the bactericidal effect on damaged biofilm structures.

We hypothesized that the magnetic nanoparticle system incorporating rhamno-lipid for EPS degradation, Ag@Fe_3_O_4_ for magnetic actuation and Ag^+^ release, and _L_-Chg_10_-teixobactin (a novel teixobactin analogue) for targeted bacterial killing, would achieve synergistic biofilm eradication. Specifically, we proposed that: (i) rhamnolipid grafting would enable EPS degradation and biofilm matrix disruption; (ii) the Ag@Fe_3_O_4_ core would provide magnetic responsiveness for enhanced biofilm penetration under RMF; and (iii) the combined antimicrobial actions of Ag^+^ and _L_-Chg_10_-teixobactin would eliminate both G^+^ and G^−^ pathogens within the biofilm, resulting in effective antibiofilm efficacy com-pared to individual components alone. Ultimately, this system progressively dismantles the biofilm matrix under RMF actuation, exposing the embedded bacterial cells, which are then effectively eliminated by the released Ag^+^ and _L_-Chg_10_-teixobactin, achieving broad-spectrum bactericidal activity and significantly enhancing the eradication efficiency of mixed-species biofilms (Scheme 1).

## 2. Materials and Methods

### 2.1. Fabrication of Ag@Fe_3_O_4_ and Rhamnolipid-Ag@Fe_3_O_4_ MNPs

The Ag@Fe_3_O_4_ was fabricated by using the versatile solvothermal reaction method according to our previously established protocol [22]. Briefly, iron (III) chloride hexahydrate (FeCl_3_·6H_2_O, ≥98.5% purity), silver nitrate (AgNO_3_, ≥99.0% purity), sodium acetate (NaAc, ≥99% purity), ethylene glycol (EG, ≥99.8% purity), and polyethylene glycol (PEG-4000, ≥99% purity) were used as precursors (Sigma-Aldrich, St. Louis, MO, USA). β-Cyclodextrin-functionalized polyethyleneimine (βCD-PEI) was synthesized according to our previously published method [22]. Rhamnolipid (RL, 90% purity) was obtained from AGAE Technologies (Corvallis, OR, USA). To graft βCD-PEI-RL polymer onto the Ag@Fe_3_O_4_ at a 3:1 mass ratio, ultrasonic dispersion (Qsonica Q500, Newtown, CT, USA, 20 kHz, 500 W) in a NaOH solution (0.5 M) was employed, followed by refrigeration at −80 °C. The RL-Ag@Fe_3_O_4_ MNPs were extracted by magnetic separation and washed thrice with deionized (DI) water.

### 2.2. Synthesis of _L_-Chg_10_-Teixobactin and Loading in RL-Ag@Fe_3_O_4_ MNPs

#### 2.2.1. _L_-Chg_10_-Teixobactin Loading Procedure

The loading of _L_-Chg_10_-teixobactin into RL-Ag@Fe_3_O_4_ MNPs was achieved through host–guest complexation with the β-cyclodextrin (βCD) moieties on the nanoparticle surface. Briefly, an _L_-Chg_10_-teixobactin stock solution in dimethyl sulfoxide (DMSO, Sigma-Aldrich, St. Louis, MO, USA) was diluted with Deionized (DI) water to obtain various working concentrations of _L_-Chg_10_-teixobactin suspension (0.03125, 0.0625, 0.125, 0.25, and 0.5 mg/mL). Next, 1.5 mL of RL-Ag@Fe_3_O_4_ MNPs suspension (2 mg/mL) was mixed with 1.5 mL of different concentrations of _L_-Chg_10_-teixobactin suspension and subjected to ultrasonication (Qsonica Q500, 20 kHz, 500 W) for 30 min to promote dispersion and initial contact, followed by incubation on an orbital shaker (Thermo Scientific MaxQ 4000, Thermo Scientific, Waltham, MA, USA, 150 rpm) at 25 °C for 12 h to facilitate the inclusion of the peptide into the hydrophobic cavity of βCD via host–guest interaction [23]. After loading, the MNPs were magnetically separated and washed three times with DI water to remove unloaded drug. The supernatant containing the loading suspension and eluent was collected [23]. The remaining amount of drug after encapsulation in MNPs was quantified by the Ultra Performance Liquid Chromatography-Mass Spectrometry using an ACQUITY UPLC H-Class system coupled with a Xevo G2-XS QTOF mass spectrometer (Waters Corporation, Milford, MA, USA) in single-ion record mode [24]. The *loading efficiency* (LE) of _L_-Chg_10_-teixobactin/RL-Ag@Fe_3_O_4_ MNPs was calculated using Equation (1) [25].

(1) $$Loading\;efficiency\left(\%\right)=\left\{\frac{\left(m_{added}-m_{unloaded}\right)}{m_{carrier}}\right\}\times 100\%$$

#### 2.2.2. Preliminary Functional Validation of Loading by Antimicrobial Activity Assay

The synthesized _L_-Chg_10_-teixobactin/RL-Ag@Fe_3_O_4_ MNPs were ultrasonically dispersed in DI water and then diluted to prepare a 400 µg/mL stock solution using the brain heart infusion (BHI) medium. Next, they were serially diluted two-fold in 96-well plates. *E. faecalis* (10^6^ CFU/mL) was inoculated into the wells at a 1:9 (*v*/*v*) ratio anaerobically for 48 h. The differences in turbidity were measured for different _L_-Chg_10_-teixobactin/RL-Ag@Fe_3_O_4_ at 100, 200, and 400 µg/mL concentrations to test the antibacterial efficacy. Each group’s optical density values were assessed at 660 nm after subtracting the background value caused by MNPs and medium. Determine the minimum MNP concentration required to inhibit bacterial growth by comparing it with the control group (0.3% *v*/*v* DMSO) [26].

### 2.3. Characterization

Various techniques were used to analyze MNPs. Scanning electron microscopy (SEM) (SU-1510/SU-4800, Hitachi, Chiyoda, Tokyo, Japan) and transmission electron microscopy (TEM) (Philips, CM100, Eindhoven, The Netherlands) were performed to analyze the morphological features of MNPs. The energy dispersion X-ray spectroscopy (EDX) analysis was performed using a scanning electron microscope equipped with a special magnetron ion sputter IXRF system. The ZetaView NP video tracking system (ParticleMetrix, PMX-120-Z GMBH, Starnberg, Germany) was used to detect the particle size and zeta potential. The analysis of Attenuated Total Reflection/Fourier Transform Infrared Spectroscopy (ATR/FTIR) (400–4000 cm^−1^ NP transmission range) was performed to characterize the chemical structure of MNPs.

### 2.4. Bacterial Culture and Mature Biofilm Formation

*E. faecalis* (ATCC 29212), *S. gordonii* (ATCC 10558), *P. gingivalis* (W 83), and *F. nucleatum* (ATCC 10953) were acquired from the American Type Culture Collection (ATCC, Manassas, VA, USA) through the central research laboratory of the Faculty of Dentistry, University of Hong Kong. The beginning of the experiment was to ensure that the bacteria grew in a logarithmic growth pattern. We mixed *E. faecalis* (10^6^ colony forming unit (CFU)/mL), *S. gordonii* (10^6^ CFU/mL), *P. gingivalis* (10^8^ CFU/mL), and *F. nucleatum* (10^8^ CFU/mL) in a 1:1:1:1 volumetric ratio.

### 2.5. Determining the Minimum Inhibitory Concentration (MIC) of _L_-Chg_10_-Teixobactin/RL-Ag@Fe_3_O_4_ MNPs and Antimicrobial Activity of Different MNPs

The MIC against the planktonic bacteria *E. faecalis*, *S. gordonii*, *F. nucleatum*, and *P. gingivalis* was detected using the broth microdilution technique [27]. An 800 µg/mL stock solution was prepared by dispersing the _L_-Chg_10_-teixobactin/RL-Ag@Fe_3_O_4_ in DI water and diluting it in brain heart infusion medium; a two-fold serial dilution was performed in 96-well plates. Bacterial inocula were added at 1:9 (*v*/*v*) ratio and anaerobically incubated (37 °C, 48 h). The optical density (OD) values of each group were measured at 660 nm and subtracted from the background value generated by MNP and medium. The MIC endpoint was the lowest nanocomposite concentration that inhibited bacterial growth compared to the control group, and no visible growth was observed after incubation [28].

The *E. faecalis*, *P. gingivalis*, *S. gordonii*, and *F. nucleatum* bacteria were exposed to 100, 200, and 400 µg/mL _L_-Chg_10_-teixobactin/RL-Ag@Fe_3_O_4_ to assess viable bacterial cell count. Bacteria grown in the BHI medium with 0.3% *v*/*v* DMSO served as a negative control [26]. Briefly, 50 µL of each bacterial suspension that underwent a 10-fold serial dilution was applied onto the solidified agar plate; the bacterial colonies growing on the plate were photographed.

The planktonic bacteria *E. faecalis*, *S. gordonii*, *F. nucleatum* and *P. gingivalis* were exposed to different MNPs (_L_-Chg_10_-teixobactin/RL-Ag@Fe_3_O_4_ MNPs and RL-Ag@Fe_3_O_4_ MNPs) at a concentration of 200 µg/mL or _L_-Chg_10_-teixobactin at 16 µg/mL (equal to the loading dosages). The bacterial suspension was introduced into the wells at a dilution ratio of 1:9 and subsequently incubated in anaerobic conditions at 37 °C for 48 h.

### 2.6. Antibiofilm Assay

The mixed bacterial biofilm cultivated under anaerobic conditions for 11 days [29] was treated with 200 µg/mL of different MNPs or 16 µg/mL _L_-Chg_10_-teixobactin for 48 h. For the _L_-Chg_10_-teixobactin/RL-Ag@Fe_3_O_4_ MNPs + rotating magnetic field (RMF) group, after 200 µg/mL of _L_-Chg_10_-teixobactin/RL-Ag@Fe_3_O_4_ MNPs were added and cultured for 46.5 h, then, the multispecies biofilm-forming confocal disks were placed on a magnetic stirrer with a magnetic intensity of 5 mT for an additional period of 1.5 h [30,31] under anaerobic conditions.

#### 2.6.1. Degradation

The EPS and bacterial cells were assessed using CLSM (FluoView-FV1000, Olympus Corporation, Tokyo, Japan) for 3D visualization of mature biofilms. EPS and bacterial cells within the multi-species biofilm were specifically labeled using Alexa Fluor 647-conjugated dextran (Thermo Fisher Scientific, Eugene, OR, USA) and SYTO-9 (Thermo Fisher Scientific, Eugene, OR, USA), respectively [32]. Three points were randomly selected from each sample for reconstructing and observing the biofilm.

After the removal of planktonic bacteria and suspended matter, the EPS in biofilms was quantified by the anthrone-sulfate method [33]. Briefly, PBS (1 mL) containing the collected biofilm from each confocal dish was transferred to a sterile centrifuge tube and centrifuged at 4000 *g* for 10 min. The pellets were dissolved in 1 mL of NaOH (0.4 mol/L) and then incubated at 37 °C for 2 h. Then, 200 µL of the supernatant was mixed with 600 µL of anthrone reagent (2 mg/mL in sulfuric acid) and incubated at 95 °C for 6 min before transferring to a new plate to measure the absorbance at 625 nm. Extracellular polymeric substance matrix loss = (extracellular polymeric substance matrix control − extracellular polymeric substance matrix treated)/extracellular polymeric substance matrix control × 100%.

#### 2.6.2. Topography and Morphology of the Biofilm Surface

The biofilms were immobilized using a 2.5% glutaraldehyde solution (Sigma-Aldrich, St. Louis, MO, USA) and subsequently underwent a gradient dehydration process. The processed biofilm samples were examined by SEM. Microscopy images were collected to observe the biofilms. Specifically, three randomly selected fields of view in each sample were analyzed under a high vacuum at accelerating voltages of 5.0 and 10.0 kV.

#### 2.6.3. Quantitative Bacterial Cell Profiling via qRT-PCR

Genomic DNA extraction from harvested biofilm specimens and planktonic cultures was performed using the QIAamp DNA Mini Kit (Qiagen, Hilden, Germany) and used for plotting standard curves. The bacteria in the biofilm samples were quantified by external standard curves. Primers listed in Table S1 [34,35] were used for SYBR Green-based assays to quantify the bacterial cells in the biofilm. Next, qRT-PCR amplification was conducted on a StepOnePlus™ Real-Time PCR System (Applied Biosystems, Waltham, MA, USA). Colony-forming equivalents (CFE) were quantified against species-specific standard curves (10^3^–10^8^ CFU/mL) constructed with *E. faecalis*, *S. gordonii*, *F. nucleatum* and *P. gingivalis*. All qRT-PCR assays were performed in three replicates.

#### 2.6.4. Biofilm Viability Profiling and Biomass Quantification

Biofilms were simultaneously stained using a combination of SYTO 9/PI (Thermo Fisher) and analyzed by CLSM. Three-dimensional reconstructions were generated using Imaris software version 10.2 (Oxford Instruments Andor, Abingdon, UK), and biofilm thickness was determined via random triplicate point sampling per specimen.

The biofilms on the confocal disks were washed three times to remove planktonic bacteria after different treatments. Then, they were stained with 1% crystal violet (CV). To dissolve the biofilms, each disk was treated with 1 mL of 95% ethanol for 15 min. The biomass of the biofilm was measured by recording the OD value of the ethanol/crystal violet solution at 590 nm through a microplate reader (Bio-Rad, Hercules, CA, USA).

### 2.7. Cytotoxicity Assessment of _L_-Chg_10_-Teixobactin/RL-Ag@Fe_3_O_4_ MNPs Toward Human Gingival Epithelial Cells (HGECs)

HGECs (CELLnTEC, Stauffacherstrasse, Switzerland) were maintained in CnT-PR medium. Fourth-passage cells were plated in a 96-well plate at 2 × 10^4^ cells per well and exposed to gradient concentrations of _L_-Chg_10_-teixobactin/RL-Ag@Fe_3_O_4_ MNPs (50–400 μg/mL) for 48–72 h. Cellular viability was quantified via CCK-8 assay following a standardized protocol.

### 2.8. Statistical Analysis

All data were derived from at least three independent experiments with consistent results, with five samples per group. Results are presented as mean ± standard deviation (SD). Significant differences were defined as * *p* < 0.05, ** *p* < 0.01, and *** *p* < 0.001. A one-way analysis of variance (ANOVA) was performed by SPSS version 19.0 (IBM, Armonk, NY, USA).

## 3. Results and Discussion

### 3.1. Synthesis of MNP Carrier: RL-Ag@Fe_3_O_4_ MNPs

**Figure 1:**
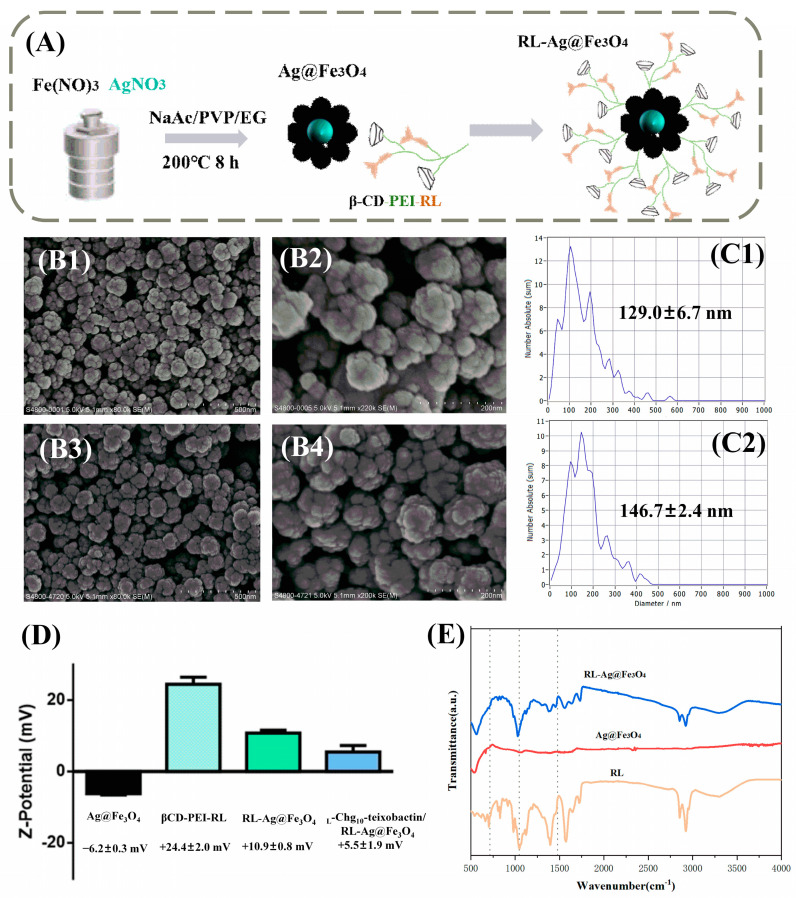
Fabrication and characterization of magnetic nanoparticles (MNPs) carrier: RL-Ag@Fe_3_O_4_. (**A**) Schematic illustration of RL-Ag@Fe_3_O_4_ MNPs synthesis; (**B**) SEM images of Ag@Fe_3_O_4_ MNPs (**B1**,**B2**) and RL-Ag@Fe_3_O_4_ MNPs (**B3**,**B4**); (**C**,**D**) “ZetaView” NP tracking system analysis of MNPs in size ((**C1**): Ag@Fe_3_O_4_ MNPs and (**C2**): RL-Ag@Fe_3_O_4_ MNPs); (**D**) Zeta potential; (**E**) ATR/FTIR spectra of MNPs.

To fabricate the carriers for anti-biofilm and magnetic nanoparticles, we assembled EPS-degrading blocks (RL) and ferromagnetic components (Ag@Fe_3_O_4_) using multiple arm PEI-βCD polymers as a connector (Figure 1A). The micromorphological features of Ag@Fe_3_O_4_ and RL-Ag@Fe_3_O_4_ MNPs were analyzed at different magnifications using SEM and TEM. The Ag@Fe_3_O_4_ nanoparticles were a core–shell structure, which aligned with the findings of a previous study [36] (Figure 1(B1,B2); Figure S1A). In contrast to the Ag@Fe_3_O_4_ MNPs, the complex RL-Ag@Fe_3_O_4_ nanoparticles did not exhibit severe aggregation or noticeable morphological changes (Figure 1(B3,B4); Figure S1B), with only a slight increase in the mean size (Ag@Fe_3_O_4_: 129.0 ± 6.7 nm; RL-Ag@Fe_3_O_4_: 146.7 ± 2.4 nm) (Figure 1(C1,C2)). The zeta potential of the Ag@Fe_3_O_4_ nanoparticle was negatively charged (−6.3 ± 0.3 mV), whereas the βCD-PEI-RL polymer exhibited a positive charge (+24.4 ± 2.1 mV). After electrostatic incorporation of βCD-PEI-RL onto Ag@Fe_3_O_4_, the resulting RL-Ag@Fe_3_O_4_ MNPs exhibited a positive charge (+10.8 ± 0.8 mV) (Figure 1D), indicating successful grafting of RL onto Ag@Fe_3_O_4_ MNPs. To further validate our findings, ATR/FTIR analyses were conducted, which revealed characteristic peaks of RL at 716, 1078, and 1462 cm^−1^ in the spectrum of RL-Ag@Fe_3_O_4_ (Figure 1E), confirming the successful grafting of RL onto Ag@Fe_3_O_4_ MNPs [37]. The EDX analysis revealed Ag and Fe conjugated with the Ag@Fe_3_O_4_ MNPs. The unique patterns of Ag and Fe in the Ag@Fe_3_O_4_ or RL-Ag@Fe_3_O_4_ are illustrated in Figure S2B,C, while Fe_3_O_4_ exhibited only a characteristic Fe pattern (Figure S2A). Quantitative results indicated that 9.2% of Ag was successfully integrated into the Fe_3_O_4_ MNPs in the form of Ag@Fe_3_O_4_ MNPs (Table S2).

### 3.2. Synthesis and Characterization of _L_-Chg_10_-Teixobactin/RL-Ag@Fe_3_O_4_ MNPs

**Figure 2:**
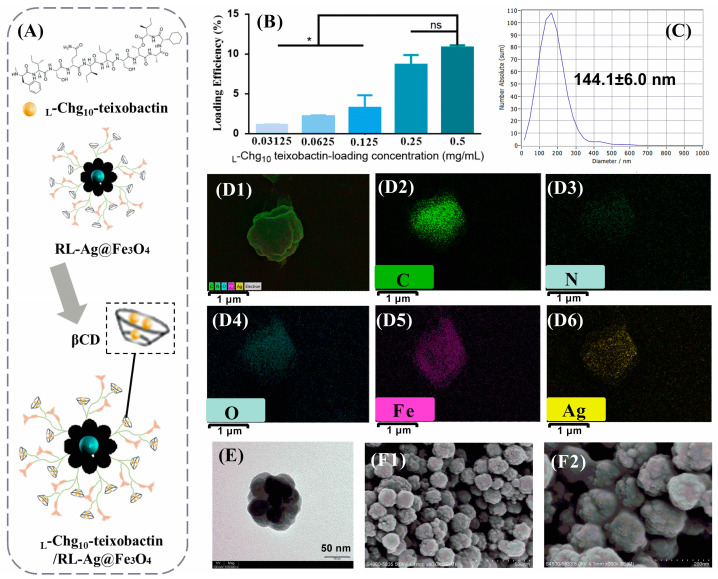
Synthesis and characterization of _L_-Chg_10_-teixobactin/RL-Ag@Fe_3_O_4_ MNPs. (**A**) Schematic illustration of loading _L_-Chg_10_-teixobactin into the RL-Ag@Fe_3_O_4_ MNPs; (**B**) The loading efficiency (LE) of the RL-Ag@Fe_3_O_4_ MNPs using different loading concentrations of the _L_-Chg_10_-teixobactin. Data are expressed from three independent experiments, and the values are presented as the mean ± standard deviation. Statistical analysis was conducted using one-way ANOVA (* *p* < 0.05, ns (non-significant): *p* ≥ 0.05); (**C**) “ZetaView” NP tracking system analysis of _L_-Chg_10_-teixobactin/RL-Ag@Fe_3_O_4_ MNPs in size; (**D1**–**D6**) Element mappings of C, N, O, Fe and Ag elements in _L_-Chg_10_-teixobactin/RL-Ag@Fe_3_O_4_ MNPs; (**E**) TEM images of _L_-Chg_10_-teixobactin/RL-Ag@Fe_3_O_4_ MNPs; (**F1**,**F2**) SEM images of _L_-Chg_10_-teixobactin/RL-Ag@Fe_3_O_4_ MNPs.

The βCD-PEI polymer, with its specific βCD structure featuring hydrophobic cavities and hydrophilic outer walls, can efficiently load the hydrophobic drug [23] (Figure 2A). It is well-known that LE is crucial in drug delivery. After loading, we determined the unloaded _L_-Chg_10_-teixobactin in both the remaining suspension and the eluate and subsequently calculated the LE of RL-Ag@Fe_3_O_4_ for _L_-Chg_10_-teixobactin. The results indicated that increasing the loading concentration of _L_-Chg_10_-teixobactin from 0.03125 to 0.25 mg/mL caused a considerable increase in the LE of RL-Ag@Fe_3_O_4_, from 1.17 ± 0.01% to 8.65 ± 1.58% (Figure 2B). However, there was no significant difference in LE between the loading concentration of 0.25 mg/mL and 0.5 mg/mL; specifically, at the loading concentration of 0.25 mg/mL, the formulation containing 3 mg of RL-Ag@Fe_3_O_4_ and 0.375 mg of _L_-Chg_10_-teixobactin achieved a LE of 8.65% (Figure 2B). The inhibitory effect of different MNPs on *E. faecalis* was also examined. The results indicated that the loading concentrations of 0.25 and 0.5 mg/mL indicated significant and consistent antimicrobial effects when the concentration of antimicrobial agents was 100, 200, or 400 µg/mL (Figure S3). Thus, we determined the loading concentration for the subsequent experiments to be 0.25 mg/mL. After loading with 0.25 mg/mL _L_-Chg_10_-teixobactin, the mean size of _L_-Chg_10_-teixobactin/RL-Ag@Fe_3_O_4_ was 144.1 ± 6.0 nm (Figure 2C), and it exhibited a charge of +5.5 ± 1.9 mV (Figure 1D). The positively charged _L_-Chg_10_-teixobactin/RL-Ag@Fe_3_O_4_ MNPs could effectively bind to the negatively charged EPS [38]. Elemental analysis mapping showed a uniform distribution of loaded elements C, N, O, Fe, and Ag in _L_-Chg_10_-teixobactin/RL-Ag@Fe_3_O_4_ MNPs (Figure 2(D1–D5)). TEM images showed the core–shell structure of _L_-Chg_10_-teixobactin/RL-Ag@Fe_3_O_4_ MNPs (Figure 2E). SEM images showed no significant morphological changes compared to RL-Ag@Fe_3_O_4_ MNPs for _L_-Chg_10_-teixobactin/RL-Ag@Fe_3_O_4_ MNPs (Figure 2(F1,F2)).

### 3.3. Antimicrobial Performance of the _L_-Chg_10_-Teixobactin/RL-Ag@Fe_3_O_4_ MNPs

**Figure 3:**
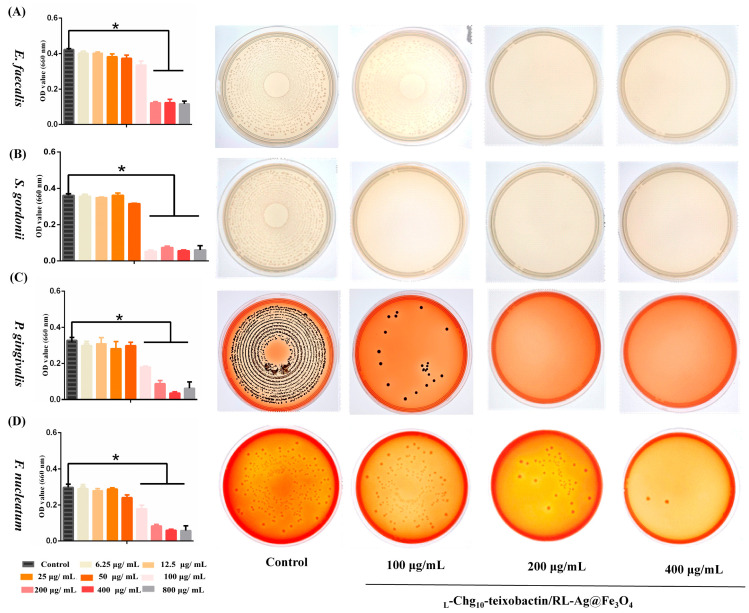
Antimicrobial performance of the _L_-Chg_10_-teixobactin/RL-Ag@Fe_3_O_4_ MNPs. (**A**–**D**) The inhibitory effect of 6.25 to 800 μg/mL _L_-Chg_10_-teixobactin/RL-Ag@Fe_3_O_4_ MNPs on bacteria growth. Bacteria treated with or without _L_-Chg_10_-teixobactin/RL-Ag@Fe_3_O_4_ MNPs (0, 100, 200, and 400 μg/mL) were cultured on agar plates ((**A**): G^+^ *E. faecalis*, (**B**): G^+^ *S. gordonii*, (**C**): G^−^ *P. gingivalis*, and (**D**): G^−^ *F. nucleatum*). Data are expressed from three independent experiments using one-way ANOVA and as the mean ± standard deviation (* *p* < 0.05).

First, the antibacterial efficacy of _L_-Chg_10_-teixobactin/RL-Ag@Fe_3_O_4_ MNPs against planktonic pathogens was assessed. The G^+^ *E. faecalis*, G^+^ *S. gordonii*, and G^−^ *P. gingivalis*, G^−^ *F. nucleatum* bacteria were exposed to concentrations ranging from 6.25 to 800 µg/mL _L_-Chg_10_-teixobactin/RL-Ag@Fe_3_O_4_ MNPs for 48 h. The inhibitory effect of _L_-Chg_10_-teixobactin/RL-Ag@Fe_3_O_4_ MNPs increased proportionally with the concentration (Figure 3A–D). The MIC of _L_-Chg_10_-teixobactin/RL-Ag@Fe_3_O_4_ on *S. gordonii* and *P. gingivalis* was 100 µg/mL, whereas the MIC of *E. faecalis* and *F. nucleatum* was 200 µg/mL (Figure 3A–D). The above-mentioned bacterial species treated with 100, 200, and 400 µg/mL _L_-Chg_10_-teixobactin/RL-Ag@Fe_3_O_4_ for 48 h were serially diluted 10-fold and cultured on agar plates for 72 h to confirm the antimicrobial effect. These results showed that 200 µg/mL _L_-Chg_10_-teixobactin/RL-Ag@Fe_3_O_4_ MNPs inhibited almost all species of bacteria. In contrast, 100 µg/mL of _L_-Chg_10_-teixobactin/RL-Ag@Fe_3_O_4_ MNPs was tolerated by *E. faecalis*, *P. gingivalis*, and *F. nucleatum* (Figure 3A–D). In addition, the CCK-8 assay was performed to evaluate the cytotoxic effects of _L_-Chg_10_-teixobactin/RL-Ag@Fe_3_O_4_ MNPs on HGECs after 48 and 72 h of exposure (Figure S4). The results demonstrated that MNPs did not exhibit cytotoxicity at concentrations up to 400 µg/mL. Thus, 200 µg/mL was selected for further investigation in subsequent experiments.

### 3.4. EPS-Degrading Biosurfactant Enhances Antibiofilm Efficacy After Grafting RL on MNPs

**Figure 4:**
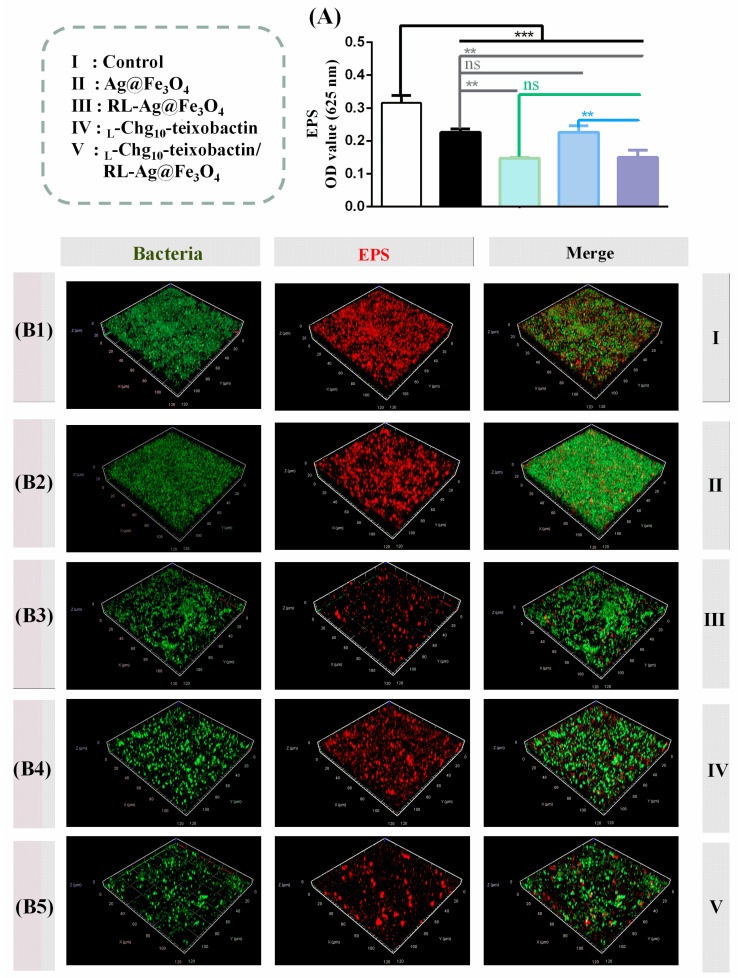
EPS-degrading biosurfactant enhances antibiofilm efficacy after grafting RL on the MNPs (**A**) The anthrone sulfuric acid quantitative analysis of EPS degrading. Data are expressed from three independent experiments using one-way ANOVA and as the mean ± standard deviation (** *p* < 0.01, *** *p* < 0.001 and ns (non-significant): *p* ≥ 0.05); (**B**) Confocal laser scanning microscopy images of EPS degrading and live bacteria ((**B1**): Control, (**B2**): Ag@Fe_3_O_4_ MNPs, (**B3**): RL-Ag@Fe_3_O_4_ MNPs, (**B4**): _L_-Chg_10_-teixobactin, (**B5**): _L_-Chg_10_-teixobactin/RL-Ag@Fe_3_O_4_ MNPs).

Novel antimicrobial technologies, such as nanoparticles and antimicrobial peptides, have limited efficacy against bacteria embedded in mature biofilms. Within the biofilm, EPS acts as a protective shield for individual bacteria, protecting them from external attacks and leading to high bacterial resistance to antibiotics [4]. Disrupting the EPS secreted by pathogenic bacteria is a challenging task that must be undertaken to combat infectious biofilm. We explored whether grafting RL (An EPS-targeting biosurfactant accelerating biofilm detachment) onto MNPs can enhance the ability to disrupt biofilms. In the control group, mature biofilm was composed of densely packed bacterial microcolonies (green) embedded in a substantial amount of EPS matrix (red) (Figure 4(B1)). Ag@Fe_3_O_4_ and _L_-Chg_10_-teixobactin alone exhibited limited effectiveness in degrading the EPS matrix of the biofilm, resulting in insignificant reductions in EPS (Figure 4(B2,B4)). In contrast, the biofilm treated with RL-Ag@Fe_3_O_4_ or _L_-Chg_10_-teixobactin/RL-Ag@Fe_3_O_4_ exhibited enhanced degradation of the EPS matrix (Figure 4(B3,B5)). The effect was more pronounced in the _L_-Chg_10_-teixobactin/RL-Ag@Fe_3_O_4_ group, which also showed bacterial dispersion simultaneously (Figure 4(B5)). RLs are amphiphilic anionic molecules composed of 1–2 rhamnose molecules and a hydrophobic tail with 1–2 fatty acid chains [39]. The hydrophobic part of the RL is embedded within the EPS, while the hydrophilic tail extends into the aqueous phase, causing the EPS to detach [40]. Quantitative analysis using the anthrone sulfuric acid method revealed that Ag@Fe_3_O_4_ and _L_-Chg_10_-teixobactin degraded 28.4% and 28.2% of the EPS, respectively. After grafting the RL, both RL-Ag@Fe_3_O_4_ and _L_-Chg_10_-teixobactin/RL-Ag@Fe_3_O_4_ exhibited enhanced EPS reduction abilities of 52.5% and 53.2%, respectively (Figure 4A). Our results showed that RL on MNPs significantly improved EPS degradation ability and loading _L_-Chg_10_-teixobactin did not considerably alter the EPS-degrading ability of RL-Ag@Fe_3_O_4_ MNPs (Figure 4A).

### 3.5. Antimicrobial Effect of _L_-Chg_10_-Teixobactin/RL-Ag@Fe_3_O_4_ and Its Compositions on the Planktonic Bacteria

**Figure 5:**
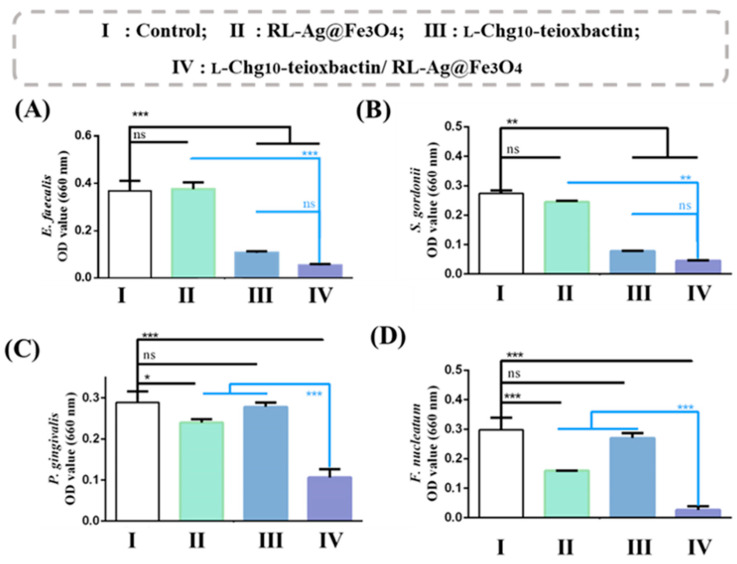
The antimicrobial effect on planktonic G^+^ and G^−^ microbial pathogens of MNPs on (**A**) G^+^ *E. faecalis*; (**B**) G^+^ *S. gordonii*; (**C**) G^−^ *P. gingivalis*; (**D**) G^−^ *F. nucleatum* growth in an anaerobic incubator at 37 °C. Data are expressed from three independent experiments using one-way ANOVA and as the mean ± standard deviation (* *p* < 0.05, ** *p* < 0.01, *** *p* < 0.001 and ns (non-significant): *p* ≥ 0.05).

The EPS-degrading biosurfactants disrupted the mechanical integrity of biofilms and induced bacterial dispersion, thereby increasing the sensitivity of biofilms to antimicrobial effects. This finding further supported the notion that EPS-targeting methods should be combined with antimicrobial agents to maximize anti-biofilm treatment efficacy. Therefore, we first investigated the antibacterial performance of _L_-Chg_10_-teixobactin/RL-Ag@Fe_3_O_4_ and its compositions (RL-Ag@Fe_3_O_4_ and _L_-Chg_10_-teixobactin) against planktonic G^+^ and G^−^ microbial pathogens. RL-Ag@Fe_3_O_4_ MNPs showed inhibitory effects on G^−^ *P. gingivalis* and G^−^ *F. nucleatum,* while no significant inhibitory effects on G^+^ *E. faecalis* and G^+^ *S. gordonii* (Figure 5A–D). Nanoparticles have been widely used in various antimicrobial studies due to their antimicrobial properties. The RL-Ag@Fe_3_O_4_ exhibited significant antimicrobial activity in this study, specifically against G^−^ but not G^+^ bacteria. This is attributed to the fact that the MNP carrier (RL-Ag@Fe_3_O_4_) primarily acted as an antimicrobial agent by releasing Ag^+^. The antimicrobial capacity of Ag^+^ depends on the outer envelope composition of the bacteria [41]. Ag^+^ demonstrates higher efficacy in targeting components such as phospholipids and lipopolysaccharides in G^−^ bacteria compared to phosphate-containing components in G^+^ bacteria [13]. In contrast to RL-Ag@Fe_3_O_4_ MNPs, _L_-Chg_10_-teixobactin exhibited more potent antimicrobial activity against G^+^ *E. faecalis* and G^+^ *S. gordonii* but did not show inhibitory effects on G^−^ *P. gingivalis* and G^−^ *F. nucleatum* (Figure 5A–D). Teixobactin can induce cell autolysis by binding and inhibiting lipid II and lipid III substrates, which are essential components of bacteria’s peptidoglycan cell wall structure [12]. In G^+^ bacteria, which lack an outer membrane, teixobactin can access the bacterial cell from the outside and bind to teichoic acids (precursors of peptidoglycan and the cell wall) [10]. However, _L_-Chg_10_-teixobactin is ineffective against G^−^ bacteria due to its inability to penetrate their robust outer membrane decorated with lipopolysaccharides [12]. Our study found that _L_-Chg_10_-teixobactin/RL-Ag@Fe_3_O_4_ MNPs grafted with EPS degradation biosurfactant and loaded with antimicrobial agents not only significantly degraded the EPS of the multispecies biofilm but also exhibited excellent antimicrobial activity against planktonic G^+^ and G^−^ bacteria (Figure 5A–D).

### 3.6. RMF-Dependent Biofilm Elimination by _L_-Chg_10_-Teixobactin/RL-Ag@Fe_3_O_4_ MNPs

**Figure 6:**
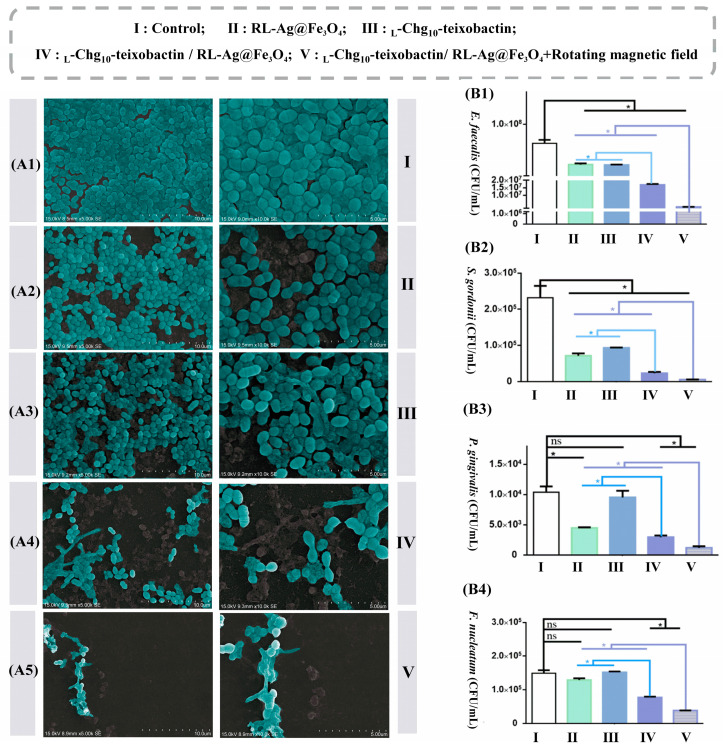
Biofilm tomography and bacterial species quantification. (**A**) SEM analyses of G^+^ *E. faecalis*, G^+^ *S. gordonii*, G^−^ *P. gingivalis*, and G^−^ *F. nucleatum* of the biofilm in (**A1**) Control, (**A2**) RL-Ag@Fe_3_O_4_ MNPs, (**A3**) _L_-Chg_10_-teixobactin, (**A4**) _L_-Chg_10_-teixobactin/RL-Ag@Fe_3_O_4_ MNPs, and (**A5**) _L_-Chg_10_-teixobactin/RL-Ag@Fe_3_O_4_ MNPs + rotating magnetic field (RMF). (**B**) The total bacteria numbers of (**B1**) G^+^ *E. faecalis*, (**B2**) G^+^ *S. gordonii*, (**B3**) G^−^ *P. gingivalis*, and (**B4**) G^−^ *F. nucleatum* were assessed by qRT-PCR. Data are expressed from three independent experiments using one-way ANOVA and as the mean ± standard deviation (* *p* < 0.05 and ns (non-significant): *p* ≥ 0.05).

**Figure 7:**
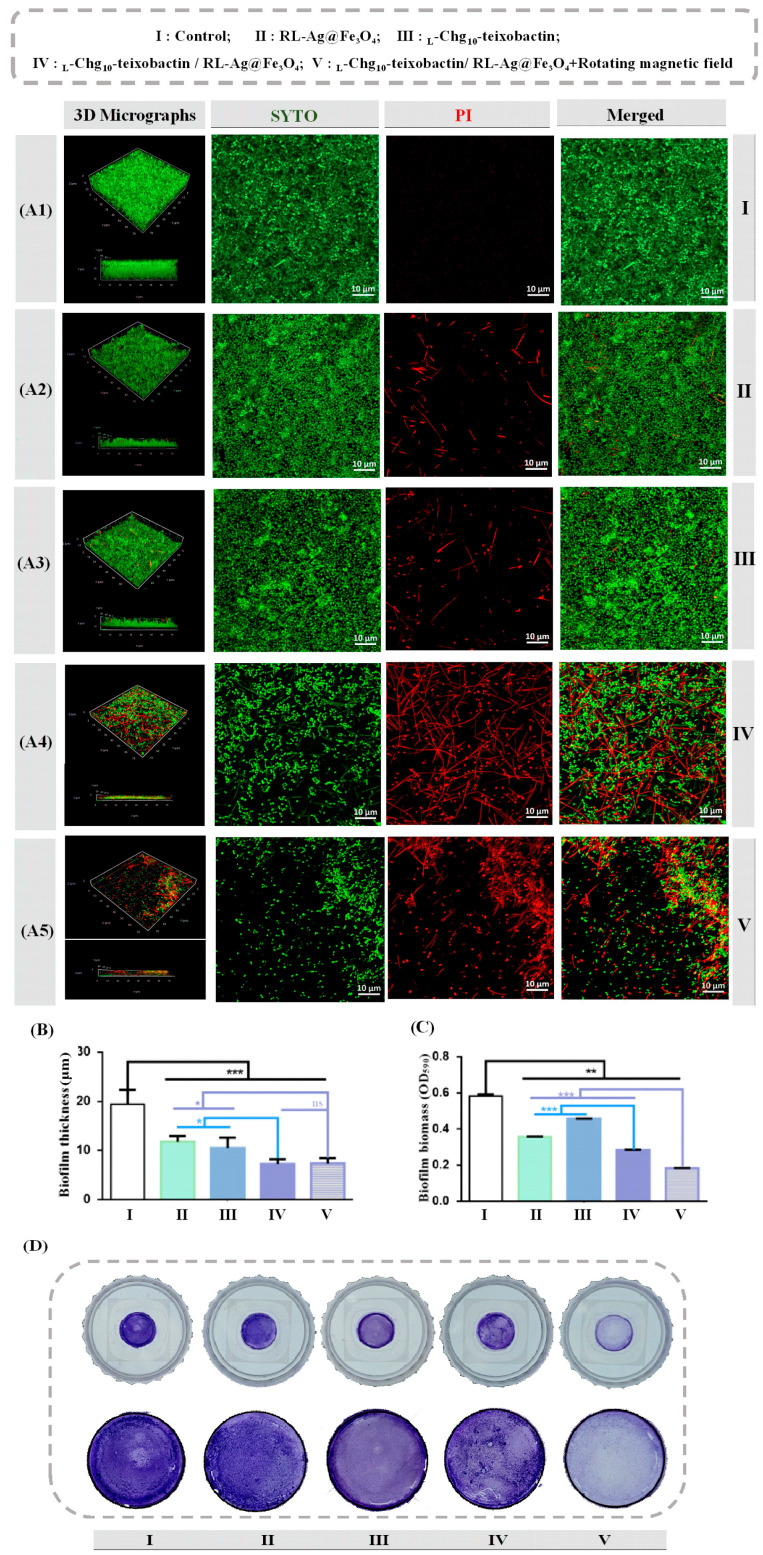
Effect of _L_-Chg_10_-teixobactin/RL-Ag@Fe_3_O_4_ MNPs on biofilm disruption. 3D and 2D CLSM images of multispecies biofilm inhibition treated by MNPs with or without a rotating magnetic field (RMF) exposure. Green (SYTO 9) and red (PI) represent live and dead bacterial cells, respectively ((**A1**) Control, (**A2**) RL-Ag@Fe_3_O_4_ MNPs, (**A3**) _L_-Chg_10_-teixobactin, (**A4**) _L_-Chg_10_-teixobactin/RL-Ag@Fe_3_O_4_ MNPs, and (**A5**) _L_-Chg_10_-teixobactin/RL-Ag@Fe_3_O_4_ MNPs+ RMF). (**B**) The average thickness of biofilm. (**C**) Biofilm biomass quantification by the OD value at 590 nm. (**D**) CV staining for remaining biomass. Data are expressed from three independent experiments using one-way ANOVA and as the mean ± standard deviation (* *p* < 0.05, ** *p* < 0.01, *** *p* < 0.001 and ns (non-significant): *p* ≥ 0.05). Scale bar: 10 µm.

After estimating the antimicrobial capacity against planktonic G^+^ and G^−^ bacterial cells, the antibiofilm performance of the _L_-Chg_10_-teixobactin/RL-Ag@Fe_3_O_4_ MNPs was investigated. It is important to note that bacteria primarily exist in biofilms rather than in a planktonic state under natural conditions. Bacteria within biofilms exhibit greater biocide tolerance than those in the planktonic state [42]. Figure 6(A1) illustrates biofilms formed by various combinations of multispecies bacteria, including G^+^ *E. faecalis*, G^+^ *S. gordonii*, G^−^ *P. gingivalis*, and G^−^ *F. nucleatum*. The mature biofilm formation was confirmed by SEM images, which showed that the disk was covered with a dense community of bacteria. The top layer consisted of a co-aggregation of *Streptococcus* species cells (Figure 6(A1)). The destruction of mature biofilm in the control group was investigated using 3D reconstructions. The multispecies biofilm exhibited a thick and well-distributed layer (19.4 ± 2.9 µm) mainly consisting of viable bacterial cells (indicated by green fluorescence) and only a few damaged bacterial pathogens (indicated by red) (Figure 7(A1)).

To assess differences in antimicrobial effects of MNPs on bacterial species between the planktonic state and biofilms, we conducted qRT-PCR analysis to quantify their abundance. The results of qRT-PCR assays suggested that the MNP carrier (RL-Ag@Fe_3_O_4_) significantly reduced the abundance of G^+^ *E. faecalis*, G^+^ *S. gordonii*, and G^−^ *P. gingivalis* by 46.71%, 69.08%, and 56.86%, respectively (Figure 6(B1–B3)), while no significant changes in abundance were observed on G^−^ *F. nucleatum* (Figure 6(B4)). However, the antimicrobial effect of RL-Ag@Fe_3_O_4_ at the same concentration (200 µg/mL) was not observed on planktonic G^+^ *E. faecalis* and G^+^ *S. gordonii* (Figure 5A,B). This difference in results was probably attributed to the dispersion of bacteria after the disruption of the EPS matrix in the biofilm, which acted as a barrier against the antimicrobial agent and maintained the stability of the biofilm. Our findings confirmed that RL-Ag@Fe_3_O_4_ can effectively degrade EPS and induce bacterial cell dispersion (Figure 4(B3)). Regarding *P. gingivalis* (G^+^ pathogens) and *F. nucleatum* (G^−^ pathogens)*,* the results indicated that RL-Ag@Fe_3_O_4_ MNPs had an antimicrobial activity against *P. gingivalis* in the plankton and biofilm states (Figure 5C and Figure 6(B3)). However, RL-Ag@Fe_3_O_4_ MNPs only showed inhibitory effects on planktonic *F. nucleatum* (Figure 5D). They did not significantly affect *F. nucleatum* in its biofilm (Figure 6(B4)), probably due to the strong tolerance of *F. nucleatum* towards antimicrobials. The upregulation of transporter-related proteins in *F. nucleatum* biofilms may explain the enhanced defense against antimicrobial compounds compared to non-attached planktonic bacterial communities [43]. Additionally, the results of qRT-PCR assays proved that _L_-Chg_10_-teixobactin significantly decreased the abundance of G^+^ *E. faecalis* and *S. gordonii* by 47.7% and 59.7%, respectively, but had no significant inhibitory effect on G^−^ *P. gingivalis* and *F. nucleatum* (Figure 6(B1–B4)); this antimicrobial effect was similar to that observed in bacterial planktonic culture (Figure 5A–D).

SEM analysis revealed distinct morphological changes in the biofilm treated with RL-Ag@Fe_3_O_4_ MNPs (Figure 6(A2)) or _L_-Chg_10_-teixobactin (Figure 6(A3)); these results indicated significant differences compared to the control group. The treated groups exhibited increased bacterial cell damage and detachment, while a substantial number of bacterial cells persisted. We found that _L_-Chg_10_-teixobactin effectively eliminated the partial chain-forming bacterial cells in the outer layer of the biofilm, spindle-shaped *F. nucleatum* appeared as a bridge between the early and late colonizing bacteria (Figure 6(A3)). Neither RL-Ag@Fe_3_O_4_ MNPs nor _L_-Chg_10_-teixobactin alone could completely disrupt the biofilm. The 3D images of the biofilms treated with RL-Ag@Fe_3_O_4_ (Figure 7(A2)) or _L_-Chg_10_-teixobactin (Figure 7(A3)) showed a higher intensity of red fluorescence, with a reduction in bacterial density and biofilm thickness (Control group: 19.4 ± 2.9 µm; RL-Ag@Fe_3_O_4_ MNPs group: 11.8 ± 1.2 µm; _L_-Chg_10_-teixobactin group: 10.5 ± 2.1 µm) (Figure 7B). The lateral view of the 3D-reconstructed biofilm indicated that treatment with either only the RL-Ag@Fe_3_O_4_ MNP carrier (which was grafted with biosurfactants capable of degrading EPS effectively) (Figure 7(A2)) or _L_-Chg_10_-teixobactin (excellent antimicrobial effect on G^−^ bacteria) (Figure 7(A3)), predominantly eliminated bacterial cells from the outer layer of the biofilm.

These results suggested that the combination of nanomaterials with EPS-degradation and antimicrobial agents, which had inhibitory effects on both G^+^ and G^−^ bacteria, disrupted the protective matrices in mature biofilm. The qRT-PCR assay further suggested that _L_-Chg_10_-teixobactin/RL-Ag@Fe_3_O_4_ MNPs decreased the levels of G^+^ *E. faecalis*, G^+^ *S. gordonii*, G^−^ *P. gingivalis*, and G^−^ *F. nucleatum* by 75.9%, 90.0%, 71.6%, and 48.2%, respectively (Figure 6(B1–B4)). The SEM analysis showed a substantial reduction in bacterial density and partial cytoplasmic leakage of biofilm treated with _L_-Chg_10_-teixobactin/RL-Ag@Fe_3_O_4_ MNPs (Figure 6(A4)). The representative CLSM images of the biofilm (Figure 7(A4)) treated with the _L_-Chg_10_-teixobactin/RL-Ag@Fe_3_O_4_ MNPs showed significant red fluorescence, along with a substantial reduction in biofilm thickness compared to treatment with RL-Ag@Fe_3_O_4_ MNPs or _L_-Chg_10_-teixobactin (_L_-Chg_10_-teixobactin/RL-Ag@Fe_3_O_4_ MNPs group: 7.3 ± 1.0 µm; RL-Ag@Fe3O4 MNPs group: 11.8 ± 1.2 µm; _L_-Chg_10_-teixobactin group: 10.5 ± 2.1 µm) (Figure 7B). From a lateral view of the 3D-reconstructed biofilm, red and yellow spots were observed within the deeper layers of the biofilms (Figure 7(A4)). This finding suggested that the strong interaction between _L_-Chg_10_-teixobactin/RL-Ag@Fe_3_O_4_ MNPs and the EPS matrix enables efficient delivery of other antimicrobial composites (Ag^+^ and _L_-Chg_10_-teixobactin), allowing them to penetrate deeper into the biofilm.

Although the potent antibacterial activity of _L_-Chg_10_-teixobactin/RL-Ag@Fe_3_O_4_ MNPs against both G^+^ and G^−^ bacteria, the SEM and CLSM showed the presence of metabolically inactive bacteria in the deeper layers of the biofilm (Figure 6(A4) and Figure 7(A4)), which could potentially result in incomplete bacterial eradication and recurrence of biofilm infection [44]. Therefore, when the biofilm was exposed to the _L_-Chg_10_-teixobactin/RL-Ag@Fe_3_O_4_ under a rotating magnetic field (RMF), we hypothesized that biofilm removal could be enhanced through the mechanical disruption of the localized biofilm structure. The results of the qRT-PCR assays indicated that the application of an RMF significantly enhanced the antimicrobial effects on both G^+^ and G^−^ bacteria compared to treatment with _L_-Chg_10_-teixobactin/RL-Ag@Fe_3_O_4_ MNPs alone. Specifically, the combination of _L_-Chg_10_-teixobactin/RL-Ag@Fe_3_O_4_ MNPs with RMF resulted in a significant reduction of 97.00% for G^+^ *E. faecalis*, 97.7% for G^+^ *S. gordonii*, 88.4% for G^−^ *P. gingivalis,* and 74.2% for G^−^ *F. nucleatum* (Figure 6(B1–B4)). The SEM images (Figure 6(A5)) showed that treatment of the biofilm with _L_-Chg_10_-teixobactin/RL-Ag@Fe_3_O_4_ MNPs and RMF resulted in the disappearance of most bacteria. The destruction of the biofilm labeled with SYTO 9/PI dual fluorescence showed that the biofilm was almost destroyed; most of the embedded bacteria were eradicated, and almost the entire biofilm disappeared (Figure 7(A5)). Although there was no significant change in the thickness of the biofilm before and after RMF application when treated with _L_-Chg_10_-teixobactin/RL-Ag@Fe_3_O_4_ MNPs (_L_-Chg_10_-teixobactin/RL-Ag@Fe_3_O_4_ MNPs: 7.3 ± 1.0 µm; _L_-Chg_10_-teixobactin/RL-Ag@Fe_3_O_4_ MNPs +RMF: 7.4 ± 1.0 µm) (Figure 7B), a notable decrease in bacterial density was observed following RMF application (Figure 7(A5)).

Furthermore, the biomass of the biofilm was quantified by CV staining, and these results were analyzed the effects of different magnetic nanocomposites on biofilm disruption. RL-Ag@Fe_3_O_4_ MNPs and _L_-Chg_10_-teixobactin maintained greater integrity than the control treatment, and they only removed 38.8% and 21.4% of biofilm biomass, respectively (Figure 7C,D). In contrast, _L_-Chg_10_-teixobactin/RL-Ag@Fe_3_O_4_ MNPs destroyed the dense biofilm structure and removed 51.4% biofilm biomass (Figure 7C,D). A considerable loss of biomass (68.5%) was found in the _L_-Chg_10_-teixobactin/RL-Ag@Fe_3_O_4_ MNPs group after RMF was applied, which destroyed the dense structure of the biofilm and caused a substantial loss of biomass (Figure 7C,D). To address the poor biofilm penetration ability of nanoparticles, various strategies have been proposed, including targeted functional modifications, porous nanostructures, and cationic modifications [45,46]. This study introduces an innovative approach by integrating the application of magnetic force with EPS-degrading biosurfactants to enhance the efficiency of biofilm removal. Our results demonstrated that the application of RMF improved the biofilm removal ability of _L_-Chg_10_-teixobactin/RL-Ag@Fe_3_O_4_ MNPs. Studies have explored magnetic nanoparticle-based strategies for biofilm eradication. Quan et al. [18] showed that Fe_3_O_4_ nanoparticles create artificial channels in *S. aureus* biofilms under static magnetic fields, enhancing MNPs penetration by approximately 4–6 fold. However, the system lacked active antimicrobial components. Elbourne et al. [30] reported that magnetic nanoparticle-functionalized liquid metal droplets achieved up to 99% removal of biofilms under RMF, yet targeted antimicrobial delivery was not incorporated. More recently, Qian et al. [31] developed “magnetic cloud bombs” using Fe_3_O_4_ nanoparticles and zeolitic imidazolate framework, demonstrating enhanced biofilm penetration under alternating magnetic fields. The present study progresses this field by integrating three complementary mechanisms into a single nanohybrid: (i) RL-mediated enzymatic EPS degradation, (ii) RMF-driven magneto-mechanical disruption, and (iii) dual antimicrobial action of Ag^+^ and L-Chg_10_-teixobactin. This synergistic combination achieved the eradication of G^+^ and G^−^ bacteria, significantly outperforming single-mechanism approaches. From a translational perspective, the RMF application represents a critical component of the therapeutic regimen. In clinical practice, this could be implemented using portable electromagnetic devices designed for intraoral use. Based on our in vitro parameters (5 mT for 1.5 h), we anticipate that a single RMF session during periodontal maintenance visits could significantly enhance biofilm disruption. However, the optimization of field strength, exposure duration, and frequency for human application will require further translational studies. Although the current nanocomposite demonstrates potent antibiofilm activity, it is important to take into account that inorganic ions commonly present in biofilm microenvironments, such as phosphate and calcium, may interact with released Ag^+^ and potentially compromise its antimicrobial efficacy. Notably, in our system, the RL-mediated degradation of EPS combined with RMF-enhanced mechanical penetration could assist in alleviating such interference by enabling a deeper and more efficient delivery of Ag^+^ to bacterial cells, thereby reducing ion entrapment within the matrix. Future studies should incorporate elemental analysis and ion-supplementation experiments to systematically evaluate these effects and further optimize the formulation for clinical translation.

## 4. Conclusions

The utilization of _L_-Chg_10_-teixobactin/RL-Ag@Fe_3_O_4_ has been found to significantly enhance the eradication of G^+^ *E. faecalis*, G^+^ *S. gordonii*, G^−^ *P. gingivalis*, and G^−^ *F. nucleatum* both in suspensions and multispecies biofilms. Incorporating RL into the system significantly improves EPS degradation within the biofilm structure, thus facilitating deeper penetration of Ag^+^ and _L_-Chg_10_-teixobactin, reducing the biomass and thickness of the biofilms. Furthermore, when an external RMF is applied, a substantial reduction in bacterial population and severe damage to the biofilm structure occur. These findings demonstrate that _L_-Chg_10_-teixobactin/RL-Ag@Fe_3_O_4_ combined with RMF has remarkable efficacy in eradicating multispecies biofilms, suggesting its promising potential for treating infectious diseases. The topical application of teixobactin analogues has expanded the potential for their clinical use. Moreover, the system is characterized by a scalable fabrication process. The solvothermal synthesis of Ag@Fe_3_O_4_ cores aligns with industrial methods, and the subsequent functionalization via electrostatic assembly and mild peptide loading is straightforward. With multiple minimally invasive delivery routes (subgingival irrigation, biodegradable films, injectable hydrogels) and the dual-action mechanism that combines targeted antimicrobial effects with RMF-enhanced physical disruption, this nanohybrid offers a translationally feasible strategy for combating biofilm-associated infectious diseases such as periodontitis.

## Data Availability

The original contributions presented in the study are included in the article, further inquiries can be directed to the corresponding authors.

## Supplementary Materials

The following supporting information can be downloaded at: https://www.mdpi.com/article/10.3390/jfb17040189/s1, Figure S1: TEM images of Ag@Fe_3_O_4_, RL- Ag@Fe_3_O_4_, and _L_-Chg_10_-teixobactin/RL-Ag@Fe_3_O; Figure S2: EDX analysis of Ag@Fe_3_O_4_, RL- Ag@Fe_3_O_4_, and _L_-Chg_10_-teixobactin/RL-Ag@Fe_3_O_4_; Figure S3: The antimicrobial effect on *E. faecalis* of _L_-Chg_10_-teixobactin/RL-Ag@Fe_3_O_4_ using different loading concentrations of _L_-Chg_10_-teixobactin; Figure S4: Cell toxicity of _L_-Chg_10_-teixobactin/RL-Ag@Fe_3_O_4_ to human gingival epithelial cells (HGECs); Table S1: Species-specific primer sequences were used in this study; Table S2: Statistical analysis of elements (Ag, Fe, C, O, and N) in MNPs by EDX.
