# Supplementary material for: Design and Development of Teixobactin Analog-Loaded Magnetic Nanocomposites for Biofilm Destruction and Pathogen Elimination
Source: J Funct Biomater. 2026 Apr 13;17(4):189. doi: 10.3390/jfb17040189 (PMC13118049; doi:10.3390/jfb17040189)
Supplement: Supplementary file 1 [file jfb-17-00189-s001.zip › jfb-4212841-supplementary.pdf]

Supporting Information:

Design and development of teixobactin analog-loaded magnetic nanocomposites for biofilm destruction and pathogens elimination

Huaxiang Lei[a,b], Ye Liang[a], Xuechen Li[d], Xiaojing Huang[b], Chengfei Zhang[a, *], Ting Zou[c, *]

[a] Restorative Dental Sciences, Faculty of Dentistry, The University of Hong Kong, Hong Kong, SAR, P. R. China.

[b] Fujian Key Laboratory of Oral Diseases & Fujian Provincial Engineering Research Center of Oral Biomaterial & Stomatological Key lab of Fujian College and University, School and Hospital of Stomatology, Fujian Medical University, Fuzhou, P. R. China.

[c] Shenzhen Clinical College of Stomatology, School of Stomatology & Shenzhen Stomatology Hospital (Pingshan), Southern Medical University, Shenzhen, P. R. China.

[d] Department of Chemistry, State Key Laboratory of Synthetic Chemistry, The University of Hong Kong, Hong Kong, SAR, P. R. China.

*** Correspondence**

Ting Zou, Shenzhen Clinical College of Stomatology, School of Stomatology & Shenzhen Stomatology Hospital (Pingshan), Southern Medical University, Shenzhen, P. R. China.

Email: zouting0818@163.com

Tel.: +755-8966-8181. Fax +755-8966-1906.

Or Chengfei Zhang, Restorative Dental Sciences, Faculty of Dentistry, The University of Hong Kong, Hong Kong, SAR, P. R. China.

E-mail: zhangcf@hku.hk.

Tel.: +852-2859-0525. Fax +852-2559-9013.

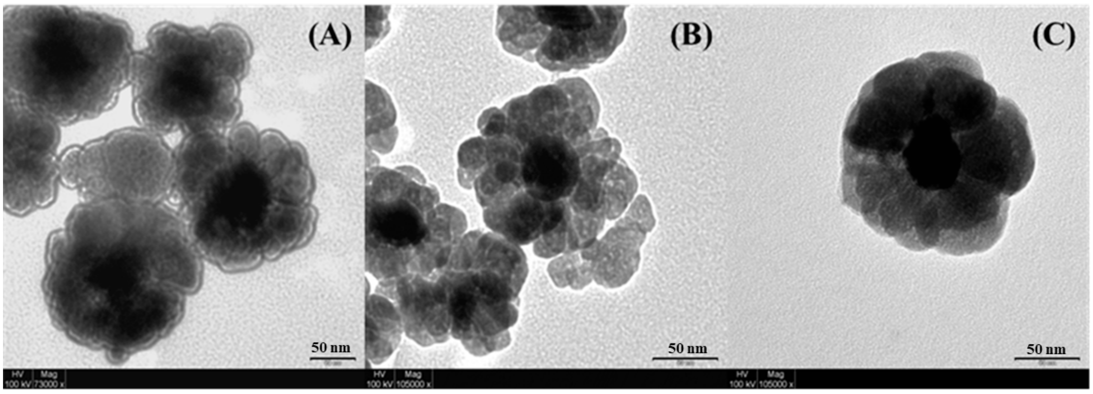

Fig. S1 TEM images of (A) Ag@Fe_3O_4 MNPs, (B) RL- Ag@Fe_3O_4 MNPs, and (C) L-Chg$_{10}$-teixobactin/RL- Ag@Fe_3O_4 MNPs.

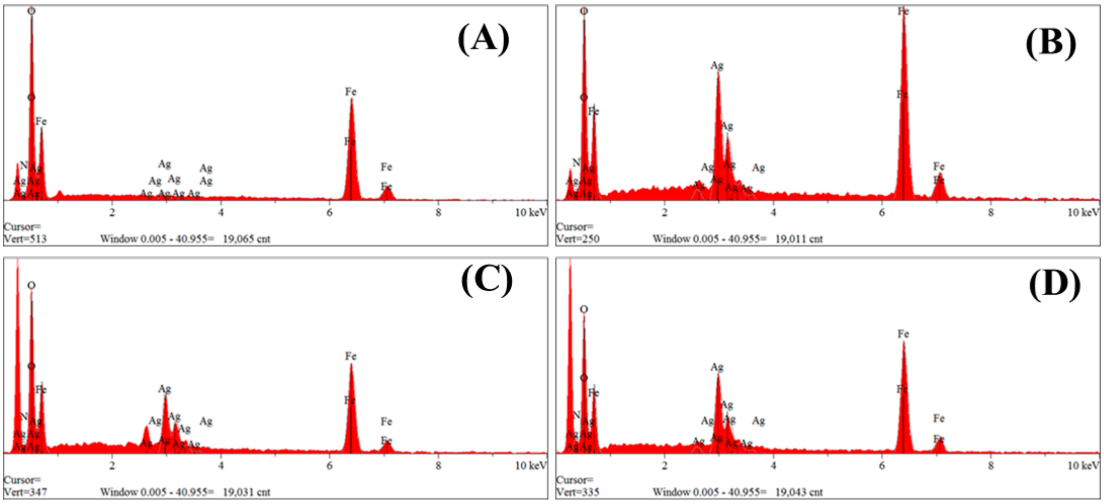

Fig. S2 Energy dispersion X-ray spectroscopic (EDX) analysis of (A) Fe_3O_4 MNPs, (B) Ag@ Fe_3O_4 MNPs, (C) RL-Ag@ Fe_3O_4 MNPs, and (D) L-Chg$_{10}$-teixobactin/RL-Ag@ Fe_3O_4 MNPs.

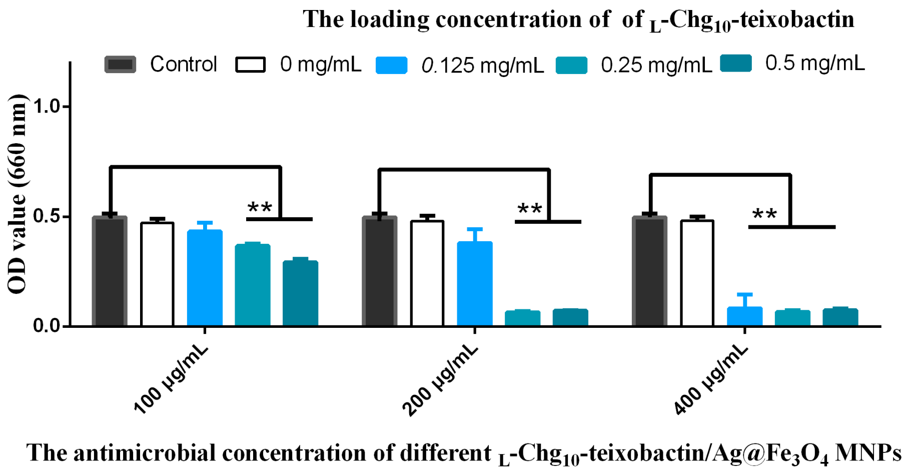

Fig. S3 The antimicrobial effect on *E. faecalis* of L-Chg10-teixobactin/RL-Ag@Fe_3O_4 MNPs using different loading concentrations of L-Chg10-teixobactin. Data are expressed from three independent experiments, and the values are presented as the mean ± standard deviation. Statistical analysis was conducted using one-way ANOVA (**P*< 0.05, ***P*< 0.01, and ****P*< 0.001);

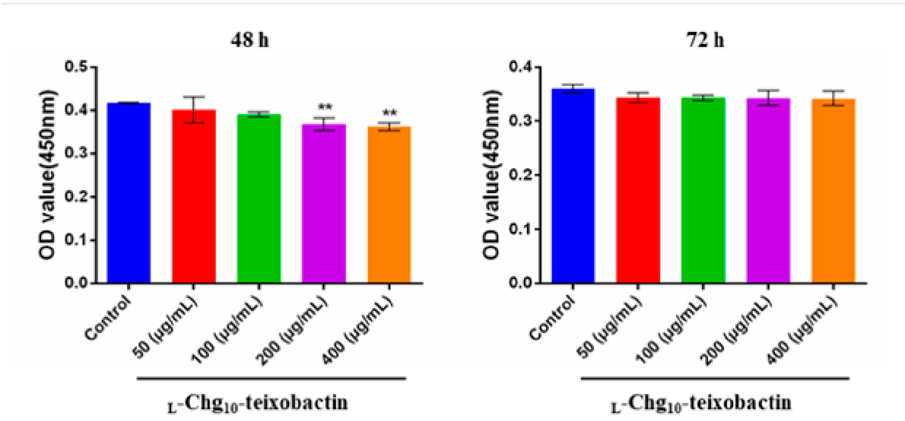

Fig. S4 Cell toxicity of 50, 100, 200 and 400 μg/mL L-Chg10-teixobactin/RL-Ag@Fe_3O_4 MNPs to human gingival epithelial cells (HGECs). Data are expressed from three independent experiments, and the values are presented as the mean ± standard deviation. Statistical analysis was conducted using one-way ANOVA (**P*< 0.05 and ***P*< 0.01);

Table S1. Species-specific primer sequences were used in this study.

Primer name	Primer sequences

	5′~3′Forward	5′~3′Reverse
E. faecalis	CGCGAACATTTGATGTGGCT	GTTGATCCGTCCGCTTGGTA
S. gordonii	GCCTTAATAGCACCGCCACT	CCATCTCTGTTGTTAGGGCGT
P. gingivalis	ACCTTACCCGGGATTGAAATG	CAACCATGCAGCACCTACATAGAA
F. nucleatum	GGATTTATTGGGCGTAAAGC	GGCATTCCTACAAATATCTACGAA

Table. S2 Statistical analysis of elements (Ag, Fe, C, O, and N) in MNPs by EDX.

MNPs / Element	Fe_3O_4	$Ag@Fe_3O_4$	$RL\text{-}Ag@Fe_3O_4$	$_{L}$-Chg_{10}-teixobactin/ $RL\text{-}Ag@Fe_3O_4$
Ag	1.0%	9.2%	4.1%	5.2%
Fe	52.9%	53.6%	33.0%	25.6%
C	14.7%	7.3%	37.0%	40.9%
O	29.9%	28.8%	24.3%	26.1%
N	1.5%	1.1%	1.8%	2.1%
